# Advances and novel developments in molecular allergology

**DOI:** 10.1111/all.14579

**Published:** 2020-09-22

**Authors:** Öykü Üzülmez, Tanja Kalic, Heimo Breiteneder

**Affiliations:** ^1^ Institute of Pathophysiology and Allergy Research Medical University of Vienna Vienna Austria

**Keywords:** allergen immunotherapy, biomarkers in allergy, hypoallergens, marker allergens, mechanisms of allergic sensitization

## Abstract

The continuous search for new allergens and the design of allergen derivatives improves the understanding of their allergenicity and aids the design of novel diagnostic and immunotherapy approaches. This article discusses the recent developments in allergen and epitope discovery, allergy diagnostics and immunotherapy. Structural information is crucial for the elucidation of cross‐reactivity of marker allergens such as the walnut Jug r 6 or that of nonhomologous allergens, as shown for the peanut allergens Ara h 1 and 2. High‐throughput sequencing, liposomal nanoallergen display, bead‐based assays, and protein chimeras have been used in epitope discovery. The binding of natural ligands by the birch pollen allergen Bet v 1 or the mold allergen Alt a 1 increased the stability of these allergens, which is directly linked to their allergenicity. We also report recent findings on the use of component‐resolved approaches, basophil activation test, and novel technologies for improvement of diagnostics. New strategies in allergen‐specific immunotherapy have also emerged, such as the use of virus‐like particles, biologics or novel adjuvants. The identification of dectin‐1 as a key player in allergy to tropomyosins and the formyl peptide receptor 3 in allergy to lipocalins are outstanding examples of research into the mechanism of allergic sensitization.

**Abbreviations**
Ab: antibody
AIT: allergen‐specific immunotherapy
BAT: basophil activation test
CCD: cross‐reactive carbohydrate determinants
CD: cluster of differentiation
CLV: clavulanic acid
CRD: component‐resolved diagnosis
CS: corticostreoid
DC: dendritic cell
EAACI: European Academy of Allergy and Clinical Immunology
FcεRI: high‐affinity IgE Fc receptor
FeNO: fractional exhaled nitric oxide
FPR: formyl peptide receptor
HDM: house dust mite
HTP: high‐throughput
Ig: Immunoglobulin
IGFALS: insulin‐like growth factor binding protein, acid labile subunit
IL: interleukin
ILCreg: regulatory innate lymphoid cells
IUIS: International Union of Immunological Societies
LPA: Luminex peptide assay
mAb: monoclonal antibody
MALDI‐MS: matrix‐assisted laser desorption/ionization time‐of‐flight mass spectrometry
miBAT: microfluidic immuno‐affinity basophil activation test
NFA: nanofluidic assay
NMR: nuclear magnetic resonance
NPT: nasal provocation test
nsLTP: nonspecific lipid transfer protein
OFC: oral food challenge
PBMC: peripheral blood mononuclear cells
PDV: *Polistes dominula* venom
PTM: post‐translational modifications
RNA: ribonucleic acid
ScFv: single‐chain variable fragment
sFcεRI: soluble isoform of high‐affinity IgE Fc receptor
sIgE: specific IgE
SLIT: sublingual immunotherapy
SPT: skin prick test
TADM: triacedimannose
TSLP: thymic stromal lymphopoietin
TUP: target‐unrelated peptide
V_H_: heavy chain variable
VLP: virus‐like particle
WHO: World Health Organization
α‐Gal: galactose‐α‐1,3‐galactose

## 1 INTRODUCTION

New technologies are changing the way research is performed in many areas of molecular allergology.^1^ Recombinant (r) allergens have influenced the development of allergy diagnosis and allergen‐specific immunotherapy (AIT) for over three decades.^2^ Although the gold rush of allergen discovery is over, gaps in our knowledge of structures, cross‐reactivities, and conformational epitopes are being constantly filled.^2^, ^3^ Precision medicine in allergology requires the identification of genes and biomarkers for diagnosis or monitoring of treatment efficacy.^4^ Such novel discoveries should be discussed, and their merits and demerits be evaluated as the research progresses. As clinical trials for the evaluation of treatment options of allergic diseases are becoming more important, the European Academy of Allergy and Clinical Immunology (EAACI) is actively involved in harmonizing and validating AIT study designs.^5^ Besides, the treatment of allergic diseases with biologics^6^ or small molecules^7^ is gaining importance. Here, we review recent advances in allergen discovery, diagnostic approaches, AIT strategies, biomarkers of allergic diseases, and mechanisms leading to allergic sensitization including an evaluation on their clinical relevance (Box 1).

Box 1 **Important recent discoveries in molecular allergology (♦ of clinical relevance)**

Discoveries about allergen cross‐reactivity

- The walnut allergen Jug r 6 is a marker allergen for clinical cross‐reactivity between walnut, hazelnut, pistachio, and sesame.♦
- Fish alpha‐parvalbumins from cartilaginous fish are hypoallergenic unlike beta‐parvalbumins from bony fish indicating a dietary alternative for patients.♦
- Venom allergens of neotropical wasps are CCD‐free, and hence, extracts of their venoms allow reliable diagnosis, unlike venom extracts from other hymenoptera species.♦

Novel technologies for the identification of linear and conformational IgE epitopes

- Linear peptides of the peanut allergen Ara h 2 were displayed on liposome nanoparticles to elucidate patient‐specific contribution of each epitope to IgE cross‐linking.
- Bead‐based assays displaying allergen‐derived sequential epitopes are sensitive and HTP tools to diagnose milk or peanut allergy.
- Chimeras based on allergen homologues or on allergens were used for mapping of conformational epitopes.

Discoveries in the field of allergen sensitization

- The food matrix contributes to the sensitization in peanut and fish allergy.♦
- A genetic predisposition for allergy to tropomyosin is caused by a single nucleotide polymorphism in the dectin‐1 gene.

Novel technologies for AIT

- Use of virus‐like particles to deliver allergens as a possible AIT strategy for peanut or HDM allergy.

## 2 ALLERGEN MOLECULES

Continuous identification and characterization of novel allergens is required for understanding the mechanisms of allergic sensitization, improving diagnostics and developing immunotherapy strategies.^1^

### 2.1 Identification of new allergens and allergenic determinants

Several novel allergens were recently accepted by the WHO/IUIS Allergen Nomenclature Sub‐Committee. An allergenic glutathione *S*‐transferase, Per a 5.0101, was extracted from the American cockroach (*Periplaneta americana*) and its immunodominant IgE epitopes were predicted using in silico approaches.^8^ Unlike the German cockroach (*Blattella germanica*) allergens Bla g 1 and 2, which are predominantly found in fecal extracts, Bla g 6, 9, and 11 are present in the whole body, and are now also recognized as major allergens.^9^ Par h 1, a pollen defensin‐like protein, was discovered from the feverfew weed (*Parthenium hysterophorus*), a so far overlooked allergen source that causes pollen‐induced allergic rhinitis.^10^ Among foods, a novel vicilin, Jug r 6, was identified from the English walnut (*Juglans regia*).^11^ The low reactivity to cartilaginous fish due to their α‐parvalbumin content indicated dietary alternatives for patients sensitized to β‐parvalbumins, major allergens of bony fish.^12^

The European paper wasp (*Polistes dominula*) is gaining importance in venom allergy research due to its invasive nature. Perez‐Riverol et al reported the absence of cross‐reactive carbohydrate determinants (CCD), which are a common cause of false‐positive results in venom allergy diagnosis of *Polistes* species.^13^ Until recently, only Pol d 5 from *P dominula* venom (PDV) was commercially available for component‐resolved diagnosis (CRD).^14^ A dipeptidyl peptidase IV, Pol d 3, was identified as another major allergen in PDV.^15^

Resistance to β‐lactam antibiotics is increasing worldwide. Consequently, novel β‐lactamase inhibitors like clavulanic acid (CLV) are co‐formulated with such antibiotics. Currently, no immunoassay is available for detecting IgE against CLV. Two possible IgE‐binding antigenic determinants of CLV were proposed as a result of protein haptenation.^16^ Moreover, two benzylpenicillin‐haptenated peptides, derived from human serum albumin, were involved in sensitization to penicillin.^17^

### 2.2 New technologies for epitope mapping and the discovery of allergens

Several official databases for searching published epitopes are available.^18^ Based on the immune epitope database,^19^ 25 times more linear than conformational epitopes are currently reported. Besides the in silico approaches, epitopes can be defined by physicochemical and biological assays. Liposomal nanoallergen display was used to analyze the individual and combined immunogenicity of eight linear epitopes from the major peanut allergen Ara h 2.^20^ For milk and peanut allergens, bead‐based epitope assays were utilized to identify patient‐specific IgE epitopes using customized peptide libraries.^21^ However, such a Luminex‐based microplate setup allows only a single antibody‐isotype measurement at a time. Ara h 1‐derived peptides displayed on phages were screened using allergic patients’ sera combined with high‐throughput (HTP) sequencing, which revealed the target‐unrelated peptide (TUP) problem.^22^ To overcome this issue, comprehensive putative TUP databases should be included for the screening. Immunodominant linear IgE epitopes of the oyster allergen Cra g 1 were also identified by a physicochemical method, the isothermal titration microcalorimetry.^23^

On the other hand, in silico approaches enable the design of chimeric proteins, where conformational epitopes of an allergen are grafted on low or nonallergenic homologues. The conformational IgE epitope profile of the Bet v 1‐related soy allergen Gly m 4 was identified by grafting its IgE‐binding areas on related nonallergenic proteins.^24^ Reversely, the olive pollen allergen Ole e 15 was mutated by introducing non‐IgE binding patches from its human homologue, which allowed the definition of conformational IgE‐binding epitopes.^25^ Yet, the studies on grafting technologies were done with small patient numbers, and correctly folded mutated chimeras are difficult to produce. Other technologies to discover epitopes such as amide hydrogen/deuterium exchange coupled with mass spectrometry, heteronuclear single quantum coherence‐NMR, monoclonal antibody (mAb) binding tests coupled with mutagenesis, and computational prediction tools, were reviewed elsewhere.^26^ These novel approaches for epitope discovery are summarized in Table 1.

**Table 1:** Novel approaches in recent discoveries of allergens and epitopes

Approach	Outcome and clinical implications
In silico allergen prediction	Discovery of 24 previously unreported allergens from Pacific oyster, filling a major gap in the management of shellfish allergic patients^26^
Liposomal nanoallergen display	Analysis of the contribution of high‐ and low‐ affinity IgE binding epitopes of Ara h 2 to the allergic response^20^
Bead‐based assays	Validation of bead‐based epitope assays for screening of food allergy^21^
	Prediction of clinical status of milk allergy, identifying a new phenotype of patients who are tolerant to fermented milk products^50^
Mapping of conformational epitopes using chimeras	Discovery of Ole e 15 epitopes and identification of two major IgE‐binding areas ^25^

Finally, HTP genome and proteome screenings combined with bioinformatics are becoming state‐of‐the‐art. Previously unreported 24 Pacific oyster allergens were identified using genome information, in silico allergen prediction and confirmation by IgE immunoblots.^27^

### 2.3 Clinically relevant cross‐reactive allergens

For the design of efficient diagnostic or immunotherapy strategies, it is crucial to understand the cross‐reactivities among allergens from various sources, which were the topic of several recent studies. The major tree nut allergens are often highly cross‐reactive. While investigating the natural Jug r 2, another walnut vicilin, Jug r 6 was discovered, which proved to be an IgE cross‐reactive marker between walnut, hazelnut, pistachio, and sesame.^11^ Moreover, examining purified natural allergens is essential for the detection of native post‐translational modifications (PTMs) such as glycosylation that may be important for IgE cross‐reactivity. Concomitant sensitization to hazelnut and peanut starts early in life, and their cross‐reactivity is possibly dominated by T‐cell epitopes of the 7S vicilins Cor a 11 and Ara h 1.^28^ Examples of nonhomologous proteins that cross‐react on the IgE and T‐cell levels are discussed in a recent review.^3^

The structure of Can f 6 was resolved, which contributed to the understanding of its cross‐reactivity with the cat allergen Fel d 4.^29^ However, conformational epitopes that selectively recognize either of these allergens are yet to be discovered to diagnose genuine dog or cat sensitization. Red meat allergy is induced by IgE specific for galactose‐α‐1,3‐galactose (α‐Gal), which is structurally similar to the blood group B‐antigen. A study of red meat allergic patients showed that blood group B individuals had almost no IgE to α‐Gal, indicating that self‐tolerance reduces the risk of developing red meat allergy.^30^ The data from a multi‐center meta‐analysis showed that the presence of B‐antigen in blood reduces the risk of developing red meat allergy.^31^

### 2.4 Structural stability of allergens

The structural stability of allergens can be an intrinsic characteristic of the proteins themselves such as heat and protease resistance or may be influenced by the presence of ligands or PTMs.^32^ Allergens, when bound to their natural ligands, may present diverse oligomeric states, like Alt a 1 from *Alternaria alternata*, that behaves differently in the presence or absence of its natural catechol‐like ligand.^33^ The tetrameric Alt a 1 holo‐form underwent receptor‐mediated endocytosis, activating the airway mucosa, while the dimeric apo‐form did not. A novel Bet v 1 ligand, phytoprostane E_1,_ was shown to inhibit the catalytic activity of cathepsin S, a cysteine protease expressed by antigen‐presenting cells, thereby protecting the allergen from degradation.^34^ nsLTPs, including Pru p 3,^35^ Mal d 3, and Cor a 8, were shown to undergo conformational changes when bound to lipid ligands, which increased their IgE recognition.^36^

Evidence is accumulating on the allergic sensitizing capacity of food matrix‐associated lipids, for instance in α‐Gal‐mediated delayed allergic reactions to red meat.^37^ The allergenic glycan α‐Gal is present in both protein and lipid extracts from red meat. However, only α‐Gal bound to lipids was transported across the intestinal cell monolayer. Food allergen passage through the gastro‐intestinal tract depends on its stability to digestion and its absorption rate. Peanut proteins are quickly absorbed into the circulation, detectable as early as 5 minutes after ingestion, and can stay immunologically intact for up to 2 days, still capable of inducing basophil activation and wheal reaction in some patients.^38^ Sensitivity of an allergen to gastro‐intestinal digestion also shapes its ability to induce oral tolerance. When the calcium‐binding residues of carp parvalbumin Cyp c 1 were mutated, the structural stability of the allergen, hence its resistance to digestion, were drastically affected.^39^ Unlike the natural allergen, the mutated form could not induce prophylactic tolerance in a mouse model of fish allergy. ^39^

Interestingly, proteins with allergenic activity were found to be relatively more abundant and stable than nonallergens in extracts from birch pollen, timothy grass, ragweed pollen, and German cockroach.^40^

## 3 DIAGNOSTIC APPROACHES

**FIGURE 1:**
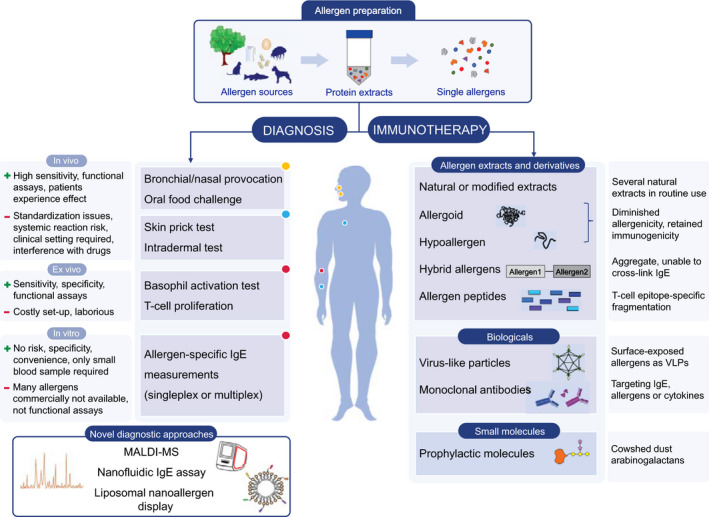
Standard and emerging strategies for diagnosis and immunotherapy of allergic diseases

Allergies are usually diagnosed by bronchial/nasal provocation test, oral food challenge, skin prick test (SPT), intradermal test, and allergen‐specific IgE (sIgE) quantification. Additional approaches such as basophil activation test (BAT), T‐cell proliferation, and CRD may help to improve the precision and comprehensiveness of the diagnostics (Figure 1).

### 3.1 Improvements in diagnosis

Component‐resolved diagnosis provides detailed data to determine sensitization profiles, to predict outcomes for persistent allergies, and to avoid allergen challenges. In a longitudinal study with egg‐allergic infants, followed up until 4 years of age, Gal d 1‐sIgE significantly increased the risk for a persistent egg allergy further in childhood.^41^ The presence of sIgE to all four known egg allergens was associated with a high risk for persistent raw‐egg allergy. CRD was also helpful to differentiate true walnut allergy from the walnut‐pecan dual allergy, reducing the need for oral food challenges.^42^ While sIgE to Jug r 1 and 4 performed best in diagnosing walnut allergy, sIgE to Jug r 2, 4, and 6 indicated a dual allergy. CRD, when performed prior to a nasal provocation test (NPT), was a safe predictive tool to diagnose allergy to dog dander.^43^ A positive NPT result demonstrated a strong positive correlation with multi‐sensitization to serum albumin, kallikrein, and lipocalin, and a negative correlation with Can f 5 (kallikrein) monosensitization. CRD may also have disadvantages, as shown when diagnosing true latex allergy. Since plant profilins and CCD‐bearing bromelains are highly cross‐reactive, CRD resulted in clinically irrelevant false‐positive results among atopic patients with pollen sensitization.^44^ For distinguishing exclusively shellfish‐sensitized patients from those sensitized to other invertebrates like molluscs, mites, and cockroaches, a decision tree based on tropomyosin and arginine kinase sequences was proposed.^45^ Such an approach, although requiring trained immunologists to determine the patients’ IgE reactivity to cross‐reactive pan‐epitopes, enables the avoidance of potential life‐threatening food challenges usually needed for diagnosis of true shellfish allergy.

Diagnosing clinically relevant food allergies caused by prior sensitization to inhalant allergens is challenging.^46^ CRD fails to diagnose clinically relevant secondary food allergies, yet BAT may deliver more reliable results for certain allergies, although its standardization is needed for clinical implementation.^47^ Clinically relevant hymenoptera allergy in patients with double sensitization to honeybee and vespid venoms was best diagnosed using BAT, thereby reducing the need for double venom immunotherapies.^48^ A post hoc analysis including 3 peanut allergy cohorts concluded that BAT performs best to predict disease severity and the threshold of consumable peanut during oral food challenge (OFC).^49^ Interestingly, a study of 70 SLIT patients showed discrepancies between the use of whole peanut extract or Ara h 2 in BAT to predict the eliciting dose in double‐blinded OFC.^50^ Unlike the whole extract, Ara h 2‐induced activation of basophils did not predict the post‐SLIT tolerance development. Another approach, the Luminex peptide assay (LPA), was used to differentiate between tolerant, whole milk‐reactive, fermented milk‐reactive and baked milk‐reactive cow's milk‐allergic subjects, based on epitope‐specific IgG4 and IgE levels.^51^

Due to unavailability in clinical practice, sIgE to Can s 3, and SPT with Can s 3‐rich hemp extract are advised for the diagnosis of Cannabis allergy.^52^ Another rare allergy is developed to corticosteroids (CS), and is commonly misdiagnosed due to excipient additives in CS preparations.^53^ An algorithm was proposed, by which several different additives in formulations could be ruled out if a preceding SPT is performed before diagnosing a genuine CS allergy.

Total allergen extracts are still widely used in routine diagnostic tests, yet potentially over‐ or underrepresented allergens are an issue. Ruethers et al tested 16 fish allergic children with 26 commercially available fish extracts in SPT.^54^ A high heterogeneity in allergen content of various extracts was reported, especially for parvalbumin and collagen. A similar issue was demonstrated for natural house dust mite (HDM) extracts, in which Der p 2, 5, 21, and 23 were underrepresented.^55^ Such examples encourage the use of predefined recombinant allergen mixtures that can be standardized.^56^

### 3.2 New approaches for diagnosis

Among the new diagnostic tools, ALEX^2®^ (Macroarray Diagnostics), a nano bead‐based platform for allergy diagnosis, correlated well with the widely used ImmunoCAP ISAC (Thermo Fisher Scientific).^57^ The nanofluidic assay (NFA) abioSCOPE^®^ allows accurate quantification of IgE at picomolar range within 5 minutes at the point‐of‐care.^58^ Validation of a chip‐based microfluidic immuno‐affinity BAT (miBAT) was compared to conventional BAT.^59^ The principle of miBAT is an initial capturing of basophils from patients’ whole blood via mobilized anti‐CD203c antibodies followed by the activation with a relevant allergen. This method has several limitations such as rather low cell purity, long analysis time, and nonspecific binding of monocytes that might result in false‐positive signals. Drug metabolite‐presenting liposomes were designed to induce the degranulation of mast cells primed with allergic patients’ sera.^60^ A direct correlation between the degranulation induced by liposomal nanoallergens in vitro and the severity of drug hypersensitivity reaction in vivo was reported.

The factors such as sex hormones, age and microbiome affects the incidence of food allergy.^61^, ^62^ Personalized approaches are hence favorable, although currently applicable only in research settings. The proof‐of‐concept of an inversed mode of CRD was reported for personalized diagnosis of cow's milk allergy.^63^ Serum IgE from allergic individuals was captured and probed with allergen extracts which allowed the analysis of eluted allergens by matrix‐assisted laser desorption/ionization time‐of‐flight mass spectrometry (MALDI‐MS).

## 4 NEW STRATEGIES FOR ALLERGEN‐SPECIFIC IMMUNOTHERAPY

The goal of AIT is clinical desensitization, meaning an increase in the threshold allergen amount needed to induce allergic symptoms.^64^ The primary outcome of successful AIT is a subjective measure such as medication and/or symptom scores.^65^ Furthermore, serum antibody and cytokine levels, and cellular activation markers are used for efficacy determination.^64^, ^65^ Conventional AIT protocols are performed over 3 years consisting of 3 phases: an initial day escalation, a buildup phase, and a maintenance period.^66^

Birch pollen allergic individuals suffer from birch pollen‐related food allergies because of sequence and structural homologies between the major birch pollen allergen Bet v 1 and related plant food allergens. Yet, birch pollen extract‐based AIT does not always improve the accompanying food allergy. In a longitudinal study, rMal d 1 sublingual immunotherapy (SLIT) successfully downregulated Mal d 1‐specific Th2 responses in birch pollen‐related apple‐allergic patients.^67^ The IgE inhibiting capacity of Mal d 1‐specific IgG antibodies correlated with the post‐SLIT clinical improvements.^68^

Adjuvants, when co‐administered with allergens in AIT, regulate the immune reaction toward Th1‐ and Treg‐type responses. Synthetic trivalent glycocluster triacedimannose was proposed as a novel adjuvant, which induced local protection when formulated with timothy grass pollen extract, triggering significantly less inflammatory cells in bronchoalveolar fluids of mice than the conventional adjuvants.^69^ In birch pollen AIT, glutaraldehyde‐treated pollen extract induced tolerance via 3‐year‐long sustained IgE‐blocking IgG4 antibodies.^70^ Peanut extract modified by reduction and alkylation and adsorbed to Al(OH)_3_ induced protective IgG antibodies in a mouse model of AIT.^71^ A depigmented HDM extract was introduced epicutaneously via laser‐generated skin micropores, omitting the need for adjuvants, and performed safe and effective in a mouse model of immunotherapy.^72^ Alternatively, allergen‐loaded microparticles decorated with neuraminidase from *Vibrio cholerae* were proven useful for targeting M‐cells for increased allergen uptake in oral immunotherapy, while inducing Th1 and Treg responses.^73^ Although performed in mouse models, these results hold promise for further development of better allergoid and adjuvant formulations for use in clinical trials.

The efficacy of recombinant allergens in AIT was first proven by Pauli et al for Bet v 1.^74^ Recombinant allergens from grass pollen, birch pollen, apple, peanut, fish, and insect venoms have been used in clinical trials in either allergen‐challenge or preventive settings. Among these, the recombinant grass pollen vaccine BM‐32 was shown to be safe and improve clinical symptoms.^75^ Various modifications can be introduced to recombinant allergens for reducing IgE reactivity while retaining T‐cell stimulatory capacity.^76^ Several novel approaches are available (Figure 1, Table 2). Hypoallergens to be used in AIT can be designed by several approaches, including producing polyvalent hybrid molecules from several allergens or introducing diverse protein modifications. The fusion of Bet v 1 and Phl p 5 is such an example, which altered their biophysical characteristics and impaired their IgE cross‐linking ability.^77^ Otherwise, destruction of both linear and conformational epitopes of Ara h 2,^78^, ^79^ and Ara h 6^79^ resulted in retained T‐cell stimulation capacity while abolishing the IgE binding.

**Table 2:** Novel strategies to induce sustained unresponsiveness or to inhibit allergic disease manifestations

Strategy	Vaccine component	Data based on	Outcome
Modified extracts, adjuvants and targeted AIT formulations	Timothy grass pollen extract adjuvanted with TADM	Mouse model	Downregulation of eosinophil and lymphocyte counts in BAL fluid and Th2 cytokine expression^69^
	Depigmented glutaraldehyde‐modified birch pollen extract	Patients’ samples	Immune response shift toward Th1, upregulation of long‐lasting IL‐10 producing specific T cells, and IgG4 production, diminished IL‐5 production^70^
	Modified peanut extract	Patients’ samples, human T‐cell line, mouse model	Decreased IgE‐binding, upregulation of specific IgG‐mediated protection against anaphylaxis, intact T‐cell proliferation capacity^71^
	Depigmented HDM extract	Patients’ samples, mouse model	Suppression of Th2 cytokines, upregulation of allergen‐specific IgG and Tregs without the need of an adjuvant^72^
	Neuraminidase of *Vibrio cholerae* for coating allergen‐loaded microparticles	Intestinal epithelial cell line, mouse model	Targeting intestinal M‐cells with neuraminidase for enhanced allergen uptake in oral AIT^73^
Allergen modifications	Phl p 5‐Bet v 1 hybrid	Patients’ samples	IgE‐reactive but hypoallergenic when compared to equimolar mixes of allergen monomers^77^
	Reduced and alkylated Ara h 2	Patients’ samples, mouse model	Reduced basophil activation, retained capacity to stimulate T‐cell proliferation ^78^
	Ara h 2 and Ara h 6 mutants with disrupted linear and conformational epitopes	Patients’ samples	Reduced basophil activation, up to 1000‐fold decrease in IgE cross‐linking capacity^79^
Peptide‐IT	Alkaline casein hydrosylate	Patients’ samples	Reduced IgE‐binding, hypoallergenic activity^80^
Virus‐like particles	Cucumber mosaic virus‐based particles expressing Ara h 1 or Ara h 2	Mouse model	Downregulation of eosinophil and mast cell infiltration of the gastro‐intestinal tract after OFC, diminished local reactions after SPT, upregulation of specific IgG‐mediated protection against anaphylaxis^84^
	Acinetobacter phage coat protein‐based particles displaying Der p 2	Mouse model	Downregulation of eosinophils in BAL fluid, upregulation of allergen‐specific IgG and alveolar macrophages^85^
	Membrane‐enveloped shielded Art v 1	Patients’ samples, mouse model	Local Treg induction correlated with downregulation of Th2 cytokines, diminished airway hyperresponsiveness upon challenge, VLPs target T cells selectively without inducing IgE^87^
mAbs and other drugs	Bet v 1‐specific ScFv	Patients’ samples	Bet v 1‐specific ScFv clone recognized homologous allergens (ie, Mal d 1, Cor a 1, Aln g 1)^90^
	Fel d 1‐specific IgG antibodies	Patients’ samples, mouse model	60% decline in nasal symptom score, 50% reduced wheal response in SPT^91^
	anti‐Fel d 1 IgY	Patients’ samples, cat model	Egg yolk products when added to cat's diet neutralized Fel d 1 in the cat, patients' allergic symptoms reduced^92^
	anti‐IL‐4Rα/IL‐5‐bispecific antibody	Mouse model	Diminished airway eosinophilia, prevention of goblet cell metaplasia^93^
	Ligelizumab	Patients’ samples, mouse model	Increased affinity to IgE, long‐lasting suppression of serum IgE levels by a single dose^95^

Food‐allergic patients may have severe reactions during AIT when intact allergens are administered. Peptide‐based approaches offer safer alternatives and may induce bystander tolerance. An edible alkaline casein hydrolysate, which preserved T‐cell immunodominant peptides, showed reduced specific antibody binding in a phase 1 study.^80^ In an animal model of dual‐allergen sensitization to Fel d 1 and ovalbumin, treatment with Fel d 1‐derived peptides alone protected mice from subsequent challenges to both cat dander extract and the unrelated allergen ovalbumin.^81^ Later, an interventional phase 3 trial was set up with the aforementioned Fel d 1‐derived peptides to asses safety and tolerability of the drug. However, the study failed due to an extraordinary high placebo effect.^82^, ^83^

Virus‐like particles (VLP), consisting of a repetitive three‐dimensional scaffold based on viral coat proteins, display allergens on the surface. Peanut‐sensitized mice were protected from anaphylaxis upon whole peanut extract challenge when treated with a VLP‐based vaccine displaying either Ara h 1 or 2.^84^ Similarly, a VLP‐based vaccine displaying Der p 2, when applied prior to sensitization, prevented the development of allergic symptoms via inducing blocking IgG antibodies in a mouse model of HDM allergy.^85^ Several VLP‐based vaccines are already commercially available for certain diseases,^86^ yet the implementation in an allergy setting is also promising. The major mugwort pollen allergen Art v 1 was used to produce allergen containing liposomes, which were hypoallergenic in a prophylactic setting when the allergens were not surface‐exposed.^87^ In the context of the hygiene hypothesis, arabinogalactans from cowshed dust extract were shown to bind human dendritic cells (DCs) and downregulate subsequent T‐cell stimulation.^88^ Such molecules could be added in formulations while designing prophylactic vaccines.

Antigen‐specific human mAbs can be produced utilizing humanized mice, phage display, by generating mAbs from immortalized human memory B cells, or by retrieving the sequence of immunoglobulin genes from single B cell clones.^89^ A combinatorial phage‐displayed single‐chain variable fragment (ScFv) library was constructed from the PBMCs of a nonallergic individual, who had been immunized with hypoallergenic Bet v 1 fragments.^90^ Bet v 1‐specific phage clones were converted into soluble ScFvs, which recognized native Bet v 1 and its homologues from alder pollen, hazelnut and apple. AIT‐induced affinity‐selected human monoclonal IgG4 prevented IgE engagement of the major cat allergen Fel d 1 in a clinical study conducted with cat‐allergic patients.^91^ Moreover, polyclonal anti‐Fel d 1 chicken antibodies produced in eggs were able to neutralize the allergen in the cat when these eggs were added to its diet.^92^ Outbred llamas are exploited for their immune responses with a wide diversity of Ab variable regions, and were used to produce bispecific antibodies against type 2 cytokines.^93^ So far, the best‐characterized mAb for allergy treatment is omalizumab. However, its use has certain limitations such as high costs and strict patient exclusion criteria.^94^ The use of ligelizumab was reported for its better inhibition of IgE‐binding to FcεRI, basophil activation, IgE production by B cells and passive systemic anaphylaxis.^95^

A novel technique was developed to sequence single cell RNA samples via 3’ barcoding, thus allowing reliable sequence recovery of T‐cell receptor and complementarity‐determining region 3.^96^ Such innovations are essential when T‐cell clonotypic responses shape the outcome of a therapy, as in AIT. Whole‐exome sequencing of B cells led to the discovery of novel convergent sequences of V_H_ regions of peanut‐specific IgE.^97^ Both IgE‐ and IgG4‐expressing B cells belonged to the same clonal family. Two unrelated peanut allergic individuals’ IgE‐expressing plasmablasts shared highly similar variable regions from heavy and light chains, holding promise for further development of mAbs competing with serum IgE.^98^

## 5 EMERGING BIOMARKERS IN ALLERGY

Allergy covers a variety of disease manifestations which all have in common the involvement of IgE. Biomarkers are needed to classify these diseases manifestations, as IgE alone is not specific enough. Precision medicine requires biomarkers for choosing the correct treatment of patients displaying various forms of allergic diseases. Recently, biomarker discovery has gained momentum.

### 5.1 Diagnostic biomarkers

Clinically applicable biomarkers for diagnosing asthma were characterized by the “SAVED” model which was reported in an EAACI position paper.^99^ Type 2‐mediated, allergy associated asthma biomarkers include cytokines such as IL‐4, IL‐5, IL‐13, TSLP, IL‐25, IL‐33 from effector lymphocytes, eosinophils and epithelial cells. Moreover, fractional exhaled nitric oxide (FeNO), exhaled volatile organic compounds, blood eosinophil count and serum IgE are among the conventional biomarkers that can be measured at the point‐of‐care. Recently, IGFALS (insulin‐like growth factor binding protein, acid labile subunit) was identified as a biomarker to differentiate allergic from nonallergic asthma subtypes.^100^ Circulating microRNAs may also help distinguishing between asthma subgroups, furthermore their expression levels correlated with disease development and clinical parameters.^101^

Eiwegger et al reviewed cellular and soluble biomarkers that are clinically relevant for food allergy.^102^ The IgE recognition and disease severity correlated with epitope abundance, diversity, and proximity on allergens in case of food allergy. More overlapping epitopes are recognized by both IgE and other isotypes such as IgG4 and IgA, as the disease progresses. In persistent egg and cow's milk allergy, increased numbers of circulating innate immune cells and downregulation of epigenetic markers, respectively, are examples of promising research to find novel targets in diagnosis and therapy.^103^, ^104^ In a metastudy including 1950 subjects, mucosal biomarkers associated with food allergy were summarized.^105^ Indicators of gut inflammation included eosinophil cationic protein, fecal calprotectin, and α1‐antitrypsin, whereas IgE and IgA indicated allergy. The soluble form of the high‐affinity IgE receptor, sFcεRI, was proposed as an accurate indicator of IgE sensitization.^106^ Finding cellular subsets and surface markers that are associated with type 2 hypersensitivity is necessary. A novel pathogenic subset of Th2 cells was associated with peanut allergy, namely the Th2A subset.^107^ Moreover, the inhibitory transmembrane molecule CD200R was identified as stably expressed surface marker on peanut allergen‐specific cells that are involved in type 2 immune responses.^108^

### 5.2 Biomarkers for monitoring AIT

Suitable biomarkers for monitoring the success of an immunotherapy depend on the disease and treatment. In allergic asthma, eosinophil counts in blood or sputum, and FeNO values are acknowledged as well‐established predictors of treatment efficacy with corticosteroids.^99^ Dipeptidyl peptidase‐4 and periostin are potential biomarkers to monitor anti‐IL‐13 monoclonal antibody treatment whereas urinary leukotriene E4 is proposed in the case of atopic asthma management with anti‐leukotrienes, yet all await validation.^99^

Sustained unresponsiveness is the goal of AIT and its monitoring is not straightforward since there are objective biomarkers to be defined for each type of allergy.^109^ An EAACI taskforce review grouped biomarkers for AIT of allergic rhinoconjunctivitis with or without asthma into seven domains.^110^ Biomarkers were categorized into serum levels of antibody subclasses, cellular activation markers, soluble messenger molecules and in vivo tests. Each domain was critically evaluated with unmet needs for promoting further clinical implementation. Regulatory types of cells with potential uses in immunotherapy follow‐up studies are continuously discovered.^66^ Emerging data indicate regulatory innate lymphoid cells (ILCregs) as a candidate biomarker cell type for monitoring the successful tolerance induction following an AIT.^111^ Although not validated yet, serum levels of vitamin D may provide an insight for natural tolerance development in egg‐allergic infants and cow's milk‐allergic children.^103^, ^112^

Omalizumab has been approved for the treatment of chronic spontaneous urticaria.^113^ Accordingly, total serum IgE levels, serum autoantibody levels and lack of basophil activation can be used to monitor the response.^114^ In a post hoc analysis of atopic diseases, absolute eosinophil counts correlated with patients’ improvements after therapeutic interventions.^115^ A critical evaluation is needed when comorbidities are present as the profile of the analyzed biomarkers may change.

## 6 NOVEL DISCOVERIES IN MECHANISMS OF ALLERGIC SENSITIZATION

Only a minority of the population develops allergic sensitization to certain proteins. The biological function of allergenic proteins, the genetic predisposition, and environmental factors are all involved in the training of the immune system and contribute to this process.^116^, ^117^ A recent review by Ozias‐Akins and Breiteneder underscored the link between the defense functions of seed storage proteins and their sensitizing capacity.^116^ Many peanut allergens display anti‐microbial/fungal properties by disrupting pathogen membranes, inhibiting pathogen growth, or they regulate cell mobility.

Allergic sensitization is also influenced by other components from allergen sources. African green monkeys developed peanut‐specific IgG but not IgE when sensitization with defatted peanut extract was performed,^118^ possibly due to the lack of peanut lipids. Moreover, peanut lipids acted as adjuvants on human keratinocytes, inducing pro‐inflammatory response and potentially aiding allergic sensitization.^119^ Similarly, low molecular weight components from bony fish extracts induced barrier damage and pro‐inflammatory cytokine release by bronchial epithelial cells.^120^

When provided the right milieu of cytokines, antibodies undergo class‐switch recombination, which decides the quality of the immune response. In peanut allergy, the class switch to IgE occurs already in the gut,^121^ supporting the emerging evidence of germinal center‐independent class‐switching.^122^ Nonetheless, the period of allergen exposure can affect the residence of IgE‐switched plasma cells and their half‐life.^123^

Sequence differences of the pattern recognition receptor dectin‐1 on respiratory epithelial cells were reported between allergic and healthy individuals.^124^ This study showed a critical role of dectin‐1 variants in regulating IL‐33 homeostasis. Other important players at the epithelial barrier are innate lymphoid 2 cells which may express CD1a and present lipid antigens.^125^ In addition, formyl peptide receptors (FPRs) from DCs were studied in the context of antigen presentation.^126^ Peptide metabolites from the allergenic lipocalins, but not from nonallergenic homologues, activated FPR3 signaling and abrogated IL‐12 production.

## 7 CONCLUSIONS

Molecular allergology has come a long way since its start in the late 1980s.^2^ While most, if not all of the important allergen types have been identified,^127^ future research will have to elucidate the molecular mechanisms of allergic sensitization and define the signal transduction cascades that ultimately lead to the production of sIgE. This will impact on prevention strategies. Furthermore, recent advances in molecular‐based allergy diagnosis should be implemented into routine diagnosis to improve patient management and to decide on personalized approaches for immunotherapy, as recently discussed by Ansotegui et al.^128^

Developing novel technologies for use in allergy diagnosis and management is crucial since it (a) aids to an in‐depth understanding of the disease, (b) collects information on advantages and disadvantages of each method for each type of allergy, and (c) encourages further research for advancing top‐notch techniques that are clinically applicable, effective and affordable. Novel treatments using next‐generation adjuvants, biologics, and small molecules are expected to advance treatment options. Allergy vaccines will be improved and fine‐tuned with the aim to achieve long‐lasting improvements. As shown in Box 2 on future research perspectives, there are many fields where molecular allergology will move forward.

Box 2 1

**Future research perspectives in molecular allergology**

Allergen characterization and modification

- Provide more structures of allergens bound with their natural ligands
- Design hypoallergenic versions of more of the clinically relevant allergens

Allergic sensitization

- Decipher signal transduction cascades induced by allergens in innate immune cells
- Identify the genetic mechanisms of allergic predisposition

Allergy diagnosis and immunotherapy

- Define the most accurate approaches to diagnose each type of allergy
- Correlate sIgE with clinical symptoms of specific types of allergy
- Evaluate and validate predictive biomarkers of allergy and markers that predict treatment response
- Design vaccine formulations for tolerance induction
- Define the role of biologics and small molecules in AIT

## CONFLICT OF INTEREST

The authors declare that they have no conflict of interest.
